# A Systematic Review of Mobile Stroke Unit Among Acute Stroke Patients: Time Metrics, Adverse Events, Functional Result and Cost-Effectiveness

**DOI:** 10.3389/fneur.2022.803162

**Published:** 2022-03-09

**Authors:** Jieyun Chen, Xiaoying Lin, Yali Cai, Risheng Huang, Songyu Yang, Gaofeng Zhang

**Affiliations:** ^1^Quanzhou First Hospital, Fujian Medical University, Fujian, China; ^2^Department of Radiology, Affiliated Hospital of Zunyi Medical University, Guizhou, China

**Keywords:** mobile stroke unit, emergency care, meta-analysis, systematic review, cost-effectiveness

## Abstract

**Background:**

Mobile stroke unit (MSU) is deployed to shorten the duration of ischemic stroke recognition to thrombolysis treatment, thus reducing disability, mortality after an acute stroke attack, and related economic burden. Therefore, we conducted a comprehensive systematic review of the clinical trial and economic literature focusing on various outcomes of MSU compared with conventional emergency medical services (EMS).

**Methods:**

An electronic search was conducted in four databases (PubMed, OVID Medline, Embase, and the Cochrane Controlled Register of Trials) from 1990 to 2021. In these trials, patients with acute stroke were assigned to receive either MSU or EMS, with clinical and economic outcomes. First, we extracted interested data in the pooled population and conducted a subgroup analysis to examine related heterogeneity. We then implemented a descriptive analysis of economic outcomes. All analyses were performed with R 4.0.1 software.

**Results:**

A total of 22,766 patients from 16 publications were included. In total 7,682 (*n* = 33.8%) were treated in the MSU and 15,084 (*n* = 66.2%) in the conventional EMS. Economic analysis were available in four studies, of which two were based on trial data and the others on model simulations. The pooled analysis of time metrics indicated a mean reduction of 32.64 min (95% confidence interval: 23.38–41.89, *p* < 0.01) and 28.26 minutes (95% CI: 16.11–40.41, *p* < 0.01) in the time-to-therapy and time-to-CT completion, respectively in the MSU. However, there was no significant difference on stroke-related neurological events (OR = 0.94, 95% CI: 0.70–1.27, *p* = 0.69) and in-hospital mortality (OR = 1.11, 95% CI: 0.83–1.50, *p* = 0.48) between the MSU and EMS. The proportion of patients with modified Ranking scale (mRS) of 0–2 at 90 days from onset was higher in the MSU than EMS (*p* < 0.05). MSU displayed favorable benefit-cost ratios (2.16–6.85) and incremental cost-effectiveness ratio ($31,911 /QALY and $38,731 per DALY) comparing to EMS in multiple economic publications. Total cost data based on 2014 USD showed that the MSU has the highest cost in Australia ($1,410,708) and the lowest cost in the USA ($783,463).

**Conclusion:**

A comprehensive analysis of current research suggests that MUS, compared with conventional EMS, has a better performance in terms of time metrics, safety, long-term medical benefits, and cost-effectiveness.

## Introduction

Acute stroke is a disease with high morbidity, disability, mortality, recurrence, and complications (1–5). The most effective treatment is revascularization within the time window (6). Advances in mobile stroke units can shorten the prehospital time due to the timeliness of thrombolytic therapy, thus improving the prognosis of acute stroke patients (7–10). Fatima et al. conducted a meta-analysis to assess the differences in time domain indexes and clinical outcomes between the MSU and regular care, discovering improved functional outcomes due to timely thrombolytic therapy (11). Therefore, scarce healthcare resources need to be effectively allocated based on more information about health economic assessments in this field. Considering the economic impact of stroke sequelae on different populations worldwide would help health authorities make reliable decisions regarding whether it deserves to implement a policy preferring MSUs.

To clarify related economic and social burden, the Global Burden of Disease study in 2016 did a careful survey that estimated the number of stroke cases worldwide to be 80.1 million, with 41.1 million women and 39 million men (12). Moreover, stroke is the second leading cause of death globally (5.5 million) after ischemic heart disease. Given the severity of the problem, the economic burden of stroke prevention and treatment represents a significant share of health care budgets in many countries (13–15). Furthermore, the cost of post-stroke care accounts for a considerable proportion of public expenditure (16). This review aims to assess various outcomes of MSUs, including time metrics, adverse events, functional results, economic estimations, compared to the conventional EMS for patients with acute stroke. We hypothesize that MSU has better clinical outcomes and is more cost-effective than EMS.

## Methods

### Search Strategy

We searched PubMed, OVID Medline, Embase, and the Cochrane Controlled Register of Trials (CENTRAL) from 1990 to 2021 for clinical trials and economic evaluations that compared MSUs with EMS for suspected patients with acute stroke. A comprehensive strategy of searching was developed by combining medical subject headings and keywords: “mobile stroke unit,” “stroke,” “ambulance,” “thrombolysis,” “functional evaluation,” and/or “cost-effectiveness” Articles published in English were included. The reference lists of the identified studies were manually searched for any missing clinical trials s or economic evaluations.

### Inclusion and Exclusion Criteria

#### Type of Study

The review included clinical trials and economic analyses.

#### Participant

Patients with acute stroke receiving the MSU or EMS treatment were included.

#### Intervention

MSU consists of prehospital thrombolysis. The MSU vehicle is a specialized ambulance equipped with a CT scanner and point-of-care laboratory and staffed by a paramedic, radiology technician, and physician specializing in neurology and emergency medicine. This vehicle is sent in response when an acute stroke is alarmed by an emergency call. Depending on the clinical symptoms, such as disabling stroke symptoms, head CT scanning and blood tests are done at the site. After being recognized as Radiologists make CT scanning interpretations, and thrombolysis is started immediately within the MSU vehicle.

#### Comparator

EMS consists of in-hospital thrombolysis. The stroke patients are taken to the hospital through emergency medical services either in the specialized stroke centers or emergency department and given thrombolysis.

Outcome. Clinical outcomes: (i) alarm to therapy decision (intravenous thrombolysis or intra-arterial recanalization), (ii) alarm to end of CT, (iii) in-hospital mortality, (iv) stroke-related or neurological events, and (v) mRs at 90 days from onset. Stroke-related or neurological events include fatal ischaemic stroke, fatal reinfarction, fatal primary intracranial hemorrhage, and fatal secondary intracranial hemorrhage. Economic outcomes: (i) MSU cost, (ii) incremental cost (difference between mean costs of intervention and mean costs of the comparator), and (iii) cost-effectiveness analysis (CEA).

Any researches involving participants younger than 18 years, interventions other than MSU, or study type other than clinical trials or economic evaluation were excluded.

### Data Extraction

The data were extracted by two authors using a structured template form. Any disagreement between the two authors was resolved by discussion.

### Statistical Analysis

Data analysis was performed through founded dichotomous outcomes like in-hospital mortality. Odds ratio (OR) is an effect size with a 95% confidence interval (CI), and study weights were estimated from the random-effects analysis. Forest plots for interested data were demonstrated. The chi-square test was conducted to evaluate whether the observed differences were heterogeneous. The *I*^2^ test was used to access inconsistencies among included studies as the percentage of variation was measured where heterogeneity was classified as 0–30% (mild), 31–50% (moderate), 51–80% (substantial), and 81–100% (considerable). Subgroup analysis was conducted when substantial to severe heterogeneity occurred. All tests were two-tailed, and a *p*-value < 0.05 was considered statistically significant. We carried out the data synthesis using narrative demonstration, with a summary of the characteristics of each included study. An overview of the combined estimation related to the MSU effect was measured for quantitative synthesis. Finally, a descriptive analysis was performed on economic outcomes. We adjusted costs by US Consumer Price Inflation Rate (based on 2014). All calculations were done with R 4.0.1 software, The R Foundation for Statistical Computing, 2004 (http://www.r-project.org). The meta-package was used to perform the meta-analyses.

### Risk of Bias

We included RCTs and non-RCTs in our study; however, no RCTs were designed as double-blind trials because of the nature of the intervention. The Revised Cochrane risk-of-bias tool (RoB 2.0) was used to appraise the methodological quality of the included studies by two reviewers independently.

## Results

### Characteristics of the Included Studies

**Figure 1:**
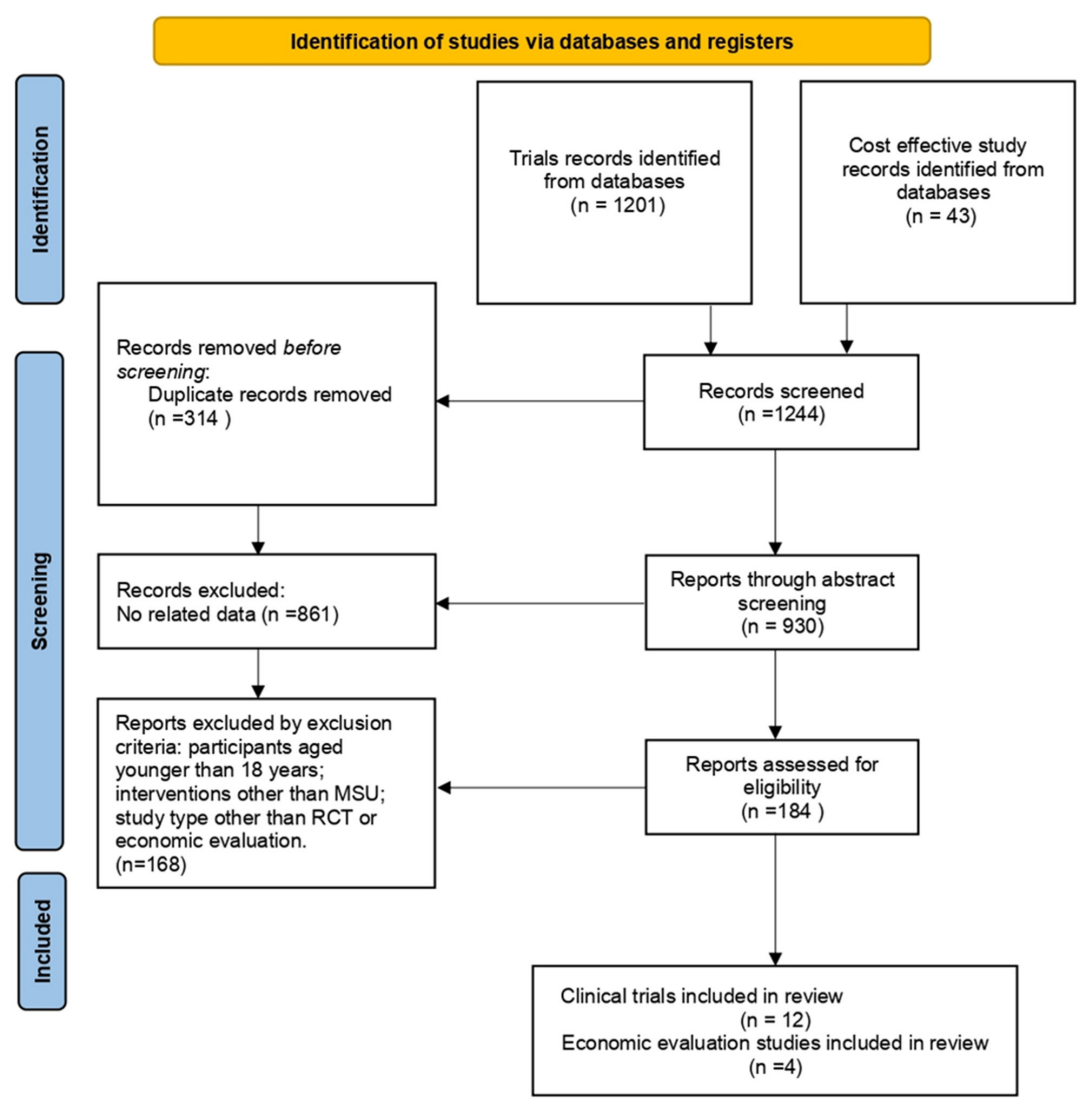
Study flowchart.

After screening 1,244 articles (Figure 1), a total of 16 articles were retrieved, including 12 clinical trials (9, 10, 17–26) and 4 economic studies (27–30). Characteristics of the included studies are described in Tables 1, 2. Most trials were done in Germany, except for five studies from the United States (Table 1). A combined total of 7,682 patients treated with MSUs were included in the trial group, and 15,084 patients treated by the conventional EMS were included in the control group (one clinical trial lacked a control group). The population's average age included in each clinical trial was around 70 years old, and more females than males. The diagnostic profile showed more patients with ischemic stroke than with hemorrhagic stroke. In addition, a complete of four economic studies were found, two based on trial data and the others on model simulations (Table 2). The earliest one was published in 2014, and the most recent one in 2021.

**Table 1:** The general characteristics of the included RCT and non-RCT studies.

**Author/year**	**Location**	**Study design**	**Number of patients in each group**	**Mean age (sd)**	**Gender (m/f)**	**Diagnosis, *n* (%)**	**Main findings**
Walter et al. (21)	Germany	RCT	Group 1 (MSU): 53 Group 2(Conventional pathway):47	Group 1: 71.2 (3.8) Group 2: 70.0 (4.5)	Group 1: 31/22 Group 2: 32/15	Group 1: Ischemic stroke: 29 (55), TIA:8(15), ICH: 4 (6), Stroke mimics: 12 (23); Group 2: Ischemic stroke:25 (53), TIA: 9(19), ICH: 7 (15), Stroke mimic: 6 (13)	MSU reduced the median time from alarm to therapy decision substantially: 35 min (IQR 31–39) vs. 76 min (63–94), *p < * 0.0001, similar gains regarding times from alarm to end of CT, and alarm to end of laboratory analysis.
Ebinger et al. (23)	Germany	RCT	Group 1 (MSU): 1,804 Group 2 (Conventional pathway):4,378	Group 1: 73.9 (15.0) Group 2: 74.2 (14.9)	Group 1: 795/1,009 Group 2: 1,970/2,408	Group 1: TIA 182 (21), Ischemic stroke 614 (70.9), ICH 45 (5.2), SAH 3 (0.3), others 22 (2.5) Group 2: TIA 643 (10.2),Ischemic stroke 2,111 (35.4), ICH 145 (2.4), SAH 11 (0.1), others 66 (0.7)	Compared with usual care, the use of MSU resulted in decreased time to treatment (15 min, 95% CI: 11–19) without an increase in adverse events (OR = 0.42, 95% CI: 0.18–1.03;*P =* 0.06).
Bowry et al. (17)	USA	RCT	Group 1 (MSU): 24 Group 2 (Conventional pathway):2	Group 1: 64	NA	Group 1: ICH:4 Seizures:4, TIA: 1, Ischemic Stroke: 11, others 6 Group 2: NA	The run-in phase of MSU provided a tPA treatment rate of 1.5 patients per week, assured us that treatment within 60 min of onset is possible, and enabled enrollment of patients on stand management weeks.
Wendt et al. (10)	Germany	RCT	Group 1 (MSU): 1,804 Group 2 (Conventional): 4,378	Group 1: 73.9 (15.0) Group 2: 74.2 (14.9)	Group 1: 646/1,158 Group 2: 1,970/2,408	Group 1: TIA 185 (10.3), Ischemic stroke 610 (33.8), Intracerebral hemorrhage 45 (2.5), Subarachnoid hemorrhage 3 (0.2), Other cerebrovascular events 23 (1.3), yNeurological non-cerebrovascular 418 (23.2), Non-neurological 520 (28.8) Group 2: TIA 461 (10.5), Ischemic stroke 1497 (34.2), Intracerebral hemorrhage 100 (2.3), Subarachnoid hemorrhage 8 (0.2), Other cerebrovascular events 44 (1.0), Neurological non-cerebrovascular 1,058 (24.2), Non-neurological 1,210 (27.6)	The triage of patients with cerebrovascular events to specialized hospitals can be improved by MSU: Two hundred forty-five of 2,110 (11.6%) patients with cerebrovascular events were sent to hospitals without Stroke Unit in conventional care when compared with 48 of 866 (5.5%; *P < * 0.01%) patients in MSU.
Parker et al. (20)	USA	RCT	Group 1 (MSU): 24 Group 2 (Conventional): NA.	NA.	NA	Group 1: ICH:4 (16.7), Seizures:3 (12.5), TIA:2 (8.3) Subdural Hematoma:1 (4.2), Time no specify:1(4.1), Ischemic Stroke:13(54.2) Group 2: NA	During an 8 week run-in phase of MSU, ≈2 patients were treated with recombinant tissue-type plasminogen activator per week, one-third within 60 min of symptom onset, with no complications.
Ebinger et al. (18)	Germany	RCT	Group 1 (MSU): 1,804 Group 2 (Conventional pathway):4,378	Group 1: 73.9 (15.0) Group 2: 74.2 (14.9)	Group 1: 795/1,009 Group 2: 1,970/2,408	Group 1: Ischemic stroke 614(70.9), TIA 182 (21), ICH 45 (5.2) Group 2: NA	Compared to conventional care, the use of MSU increases the percentage of patients receiving thrombolysis within the golden hour (62 of 200 patients [31.0%] vs. 16 of 330 [4.9%];*P < * 0.01).
Kunz et al. (25)	Germany	Non-RCT	Group 1 (MSU): 305 Group 2 (Conventional pathway): 353	Group 1: 70.7 (11.9) Group 2: 70.2 (10.4)	Group 1: 159/146 Group 2: 223/130	NA	Compared with conventional care, adjusted odds ratios (ORs) for MSU for the primary outcome (OR 1.40, 95% CI 1.00–1.97; *p =* 0.052) were not significant. Intracranial hemorrhage (*p =* 0.27) and 7-day mortality (*p =* 0.23) did not diff er significantly between treatment groups.
Taqui et al. (26)	USA	RCT	Group 1 (MSU): 100 Group 2 (Conventional pathway):53	Group 1: 63.7(17.0) Group 2: 66.3(16.0)	Group 1: 46/54 Group 2: 23/30	NA	There was a significantreduction of median alarm-to-CT scan completion times (33 min MSTU vs. 56 min con-trols, *p* < 0.0001), median alarm-to-thrombolysis times (55.5 min MSTU vs. 94 min controls, *p* < 0.0001), median door-to-thrombolysis times (31.5 min MSTU vs. 58 min controls, *p < * 0.001), and symptom-onset-to-thrombolysis times (97 min MSTU vs. 122.5 min controls, *p < * 0.04). Sixteen patients evaluated on MSTU received thrombolysis, 25% of whom received it within 60 min of symptom onset.
Kummer et al. (24)	USA	Non-RCT	Group 1 (MSU): 66 Group 2 (Conventional pathway):19	Group 1: 77.2 (16.2) Group 2: 71.6 (11.3)	Group 1: 28/38 Group 2: 10/9	Group 1: Ischemic stroke 31 (47.0), ICH 5 (7.6), TIA 3 (4.5) and stroke mimics 27 (40.9) Group 2: Ischemic stroke 9 (47.4), ICH 1 (5.3), TIA 2 (10.5), and stroke mimics 6 (31.6)	Compared with patients receiving conventional care, patients receiving MSU care were significantly more likely to be picked up closer to a higher mean number of designated stroke centers in a 2.0-mile radius (4.8 vs. 2.7, *P =* 0.002). In multivariable analysis, MSU care was associated with a mean decrease in dispatch-to-thrombolysis time of 29.7 min (95% CI, 6.9–52.5) compared with conventional care.
Helwig et al. (19)	Germany	RCT	Group 1 (MSU): 63 Group 2 (Conventional pathway): 53	Group 1: 75 (11.0) Group 2: 74 (11.0)	Group 1: 27/36 Group 2: 17/36	Group 1: Ischemic stroke 32 (50.8), ICH 8 (12.7), TIA 17 (27.0) and stroke mimics 6 (9.5) Group 2: Ischemic stroke 39 (73.6), ICH 8 (15.1), TIA 4 (7.5), and stroke mimics 2 (3.8)	MSU-based management enables accurate triage decisions for 100%, although patient outcomes were not significantly different.
Grotta et al. (22)	USA	Non-RCT	Group 1 (MSU): 886 Group 2 (Conventional pathway): 629	Group 1: 67 (3.6) Group 2: 65 (3.6)	Group 1: 432/454 Group 2: 341/288	NA	Among the patients eligible for t-PA, 55.0% in the MSU group and 44.4% in the EMS group had a score of 0 or 1 on the modified Rankin scale at 90 days.
Ebinger et al. (23)	Germany	RCT	Group 1 (MSU): 749 Group 2 (Conventional pathway): 749	Group 1: 73 (13) Group 2: 74 (13)	Group 1: 403/346 Group 2: 417/377	Group 1: Ischemic stroke 625 (83.4), TIA 124 (16.6) Group 2: Ischemic stroke 663 (83.5), TIA 131 (16.5)	The dispatch of MSU, compared with conventional ambulances alone, was significantly associated with lower global disability at 3 months (common OR for worse mRS, 0.71; 95% CI, 0.58–0.86;*P < * 0.001).

*ICH, intracranial hemorrhage; RCT, randomized controlled trial; MSU, mobile stroke unit; SAH, subarachnoid hemorrhage; TIA, transient ischemic attack; tPA, tissue plasminogen activator; NA, not applicable*.

**Table 2:** The general characteristics of the included economic studies.

**Author**	**Sources**	**Location**	**Duration**	**Study design**	**Year**	**Cost of study intervention $**	**Net cost of intervention $**	**Cost of study control $**	**Incremental cost $**	**Outcomes**	**Cost-saving $**
Dietrich et al. (27)	Current wage agreements of the German public service	Germany	1 year	Trial-based	2014	1,207,753	NA	NA	236,568	Benefit-cost ratio: 1.96	463,124
Gyrd-Hansen et al. (28)	Berlin fire department and Charité hospital Official human resources tables	Germany	10.5 months	Trial-based	2015	1,410,708^a^	947,767	NA	NA	Cost-effectiveness ratio: 31,911 per QALY	481,482
Kim et al. (29)	MSU financial and patient tracking reports and related databases.	Australia	1 year	Model-based	2019	1,881,331^a^	1,736,617	NA	NA	Cost-effectiveness ratio: 38,731 per DALY	295,033
Reimer et al. (30)	Bureau of Labor and Statistics Peer-reviewed published literature.	USA	15 months	Model-based	2020	783,463^a^	NA	785,869	70,613	NA	NA

*$, US Dollar; benefit-cost ratio, cost saving/incremental cost; cost-effectiveness ratio, net cost/outcomes.*

*^a^Adjusted by US Consumer Price Inflation Rate (based on 2014)*.

### Quality Assessment

**Figure 2:**
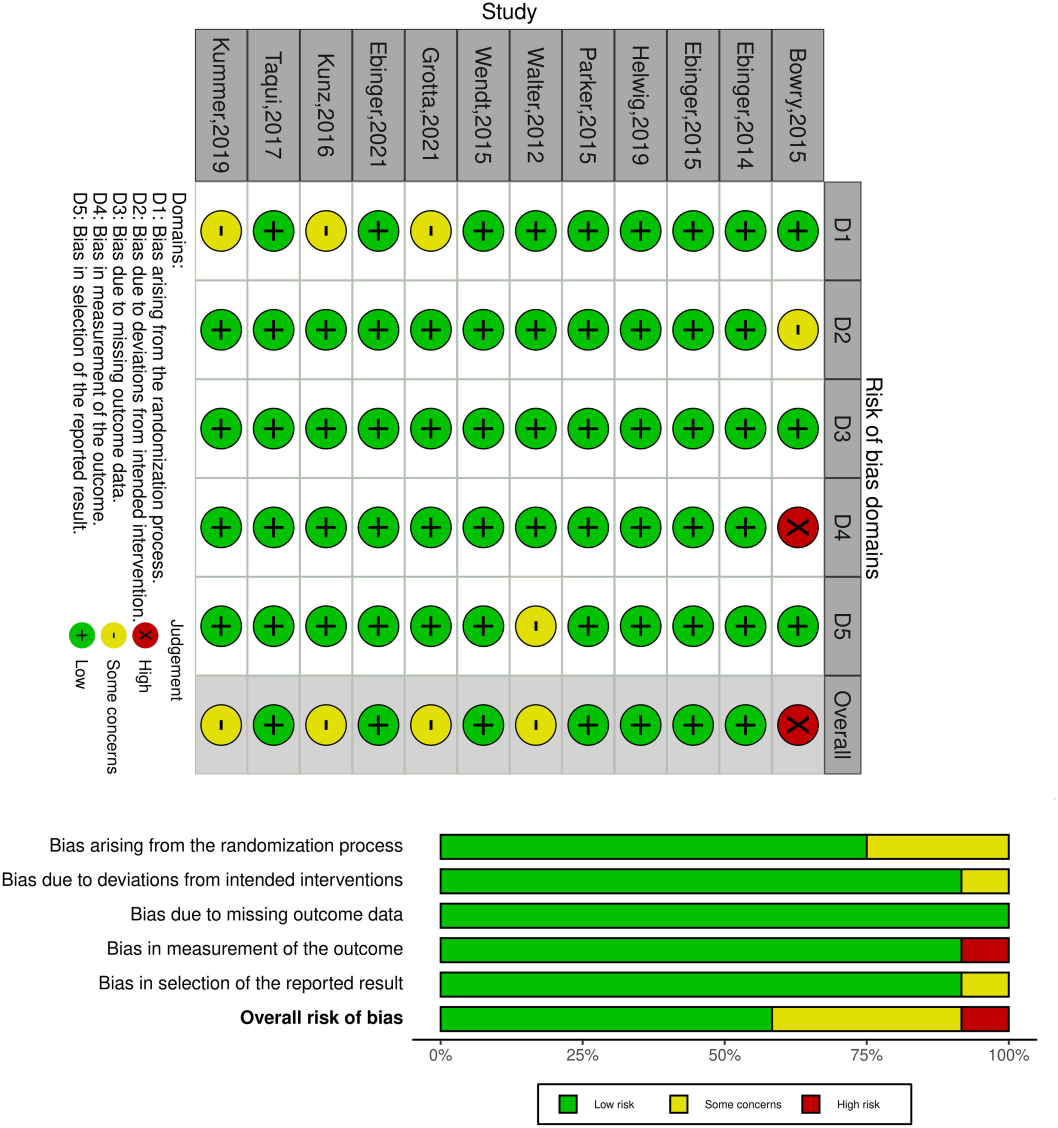
Bias risk assessment for inclusion in the study.

Selection, performance, detection, attrition, and reporting bias were assessed as high, low, or some concerns. As shown in Figure 2, all RCTs were considered high risk of bias. The trial dates ranged from 2014 to 2021, of which seven were assessed low risk of bias, with five moderate and one high.

### Time Metrics

**Figure 3:**
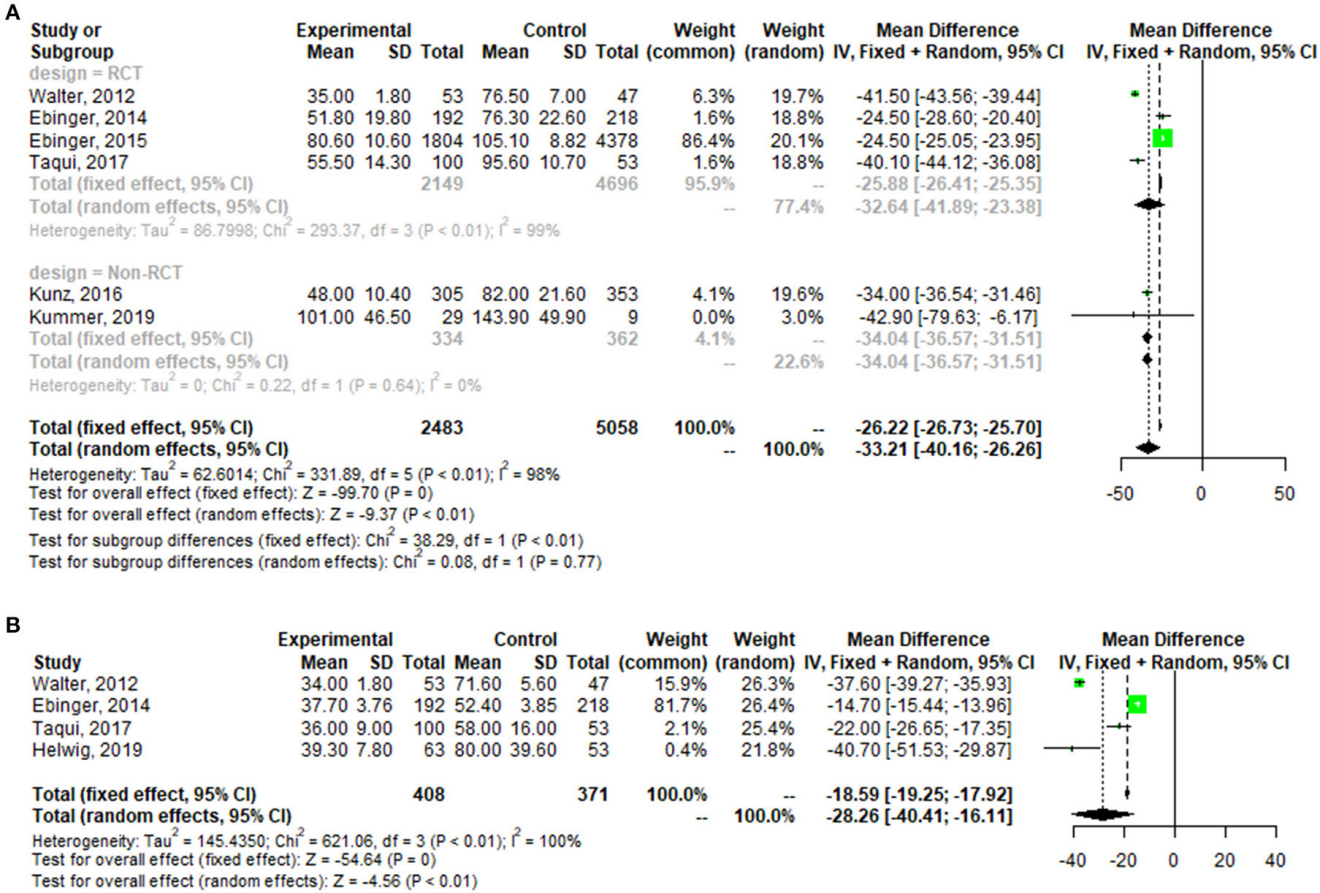
Forest plot for change in the time from alarm to therapy decision **(A)**, and the time from alarm to CT completion **(B)**.

**Figure A1:**
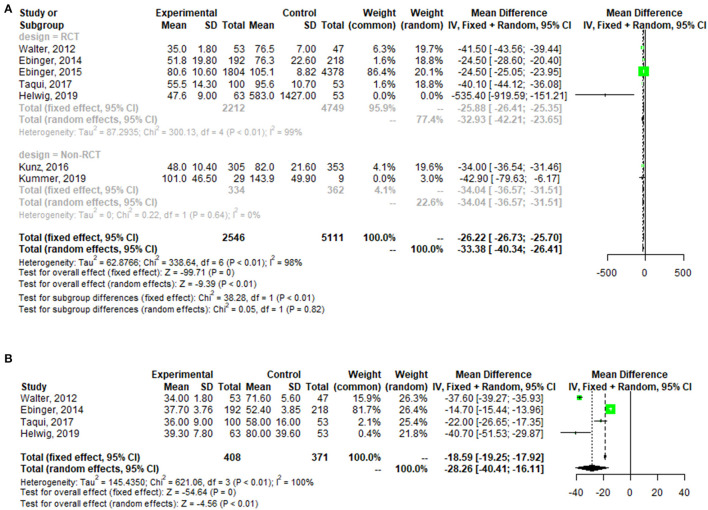
Forest plot for change in the time from alarm to therapy decision [adding Helwig (19)] **(A)**, and the time from alarm to CT completion **(B)**.

A pooled analysis of the time from alarm to therapy decision for all patients showed that the MSU group had a mean reduction of 32.64 min (95% confidence interval: 23.38–41.89, *p* < 0.01; one outlier was excluded (see Figure A1) compared with the control group. The pooled analysis of patient time from alarm to CT completion presented similar results, with a mean reduction of 28.26 min (95% confidence interval: 16.11–40.41, *p* < 0.01) for patients in the MSU group compared to the regular care group (Figure 3).

### Adverse Events

**Figure 4:**
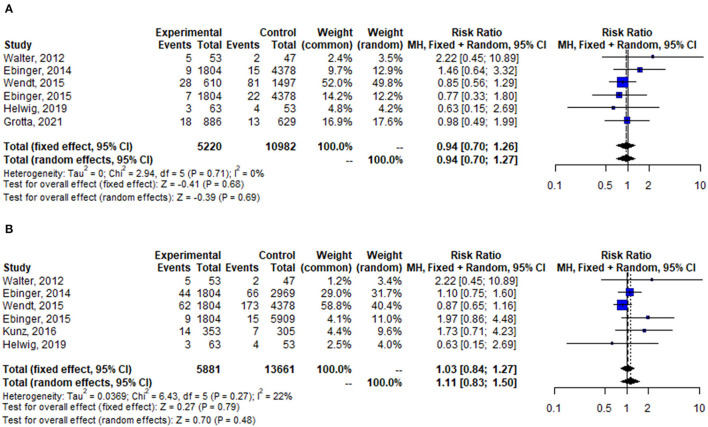
Forest plot for change in stroke-related or neurological events **(A)**, and in-hospital mortality **(B)**.

Pooled analysis of stroke-related or neurological events was not statistically different between the two groups (OR = 0.94, 95% CI: 0.70–1.27, *p* = 0.69), it was the same at in-hospital mortality (OR = 1.11, 95% CI: 0.83–1.50, *p* = 0.48) (Figure 4).

### mRS

**Figure 5:**
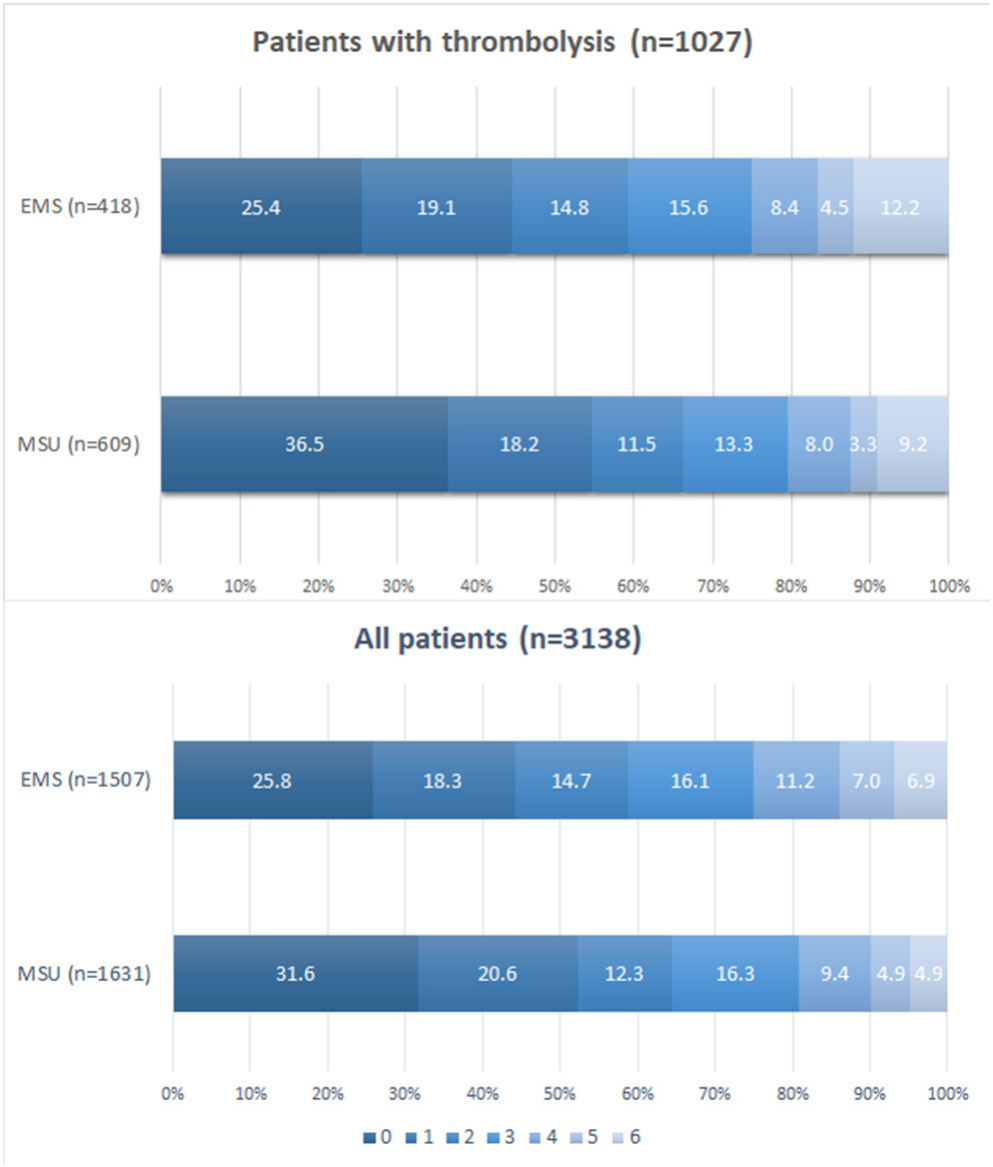
Pooled analysis of scores on mRS at 90 days.

Four of the twelve clinical studies reported mRs at 90 days from onset. Figure 5 discovered the distribution of mRS by treated population at 90 days from onset. Pooled data showed that the proportion of patients with mRS 0–2 at 90 days from onset was higher in the MSU group than in the EMS group among the thrombolysis population and all populations (*p* < 0.05).

### Cost-Effectiveness

The cost-effectiveness outcomes of the included economic studies are shown in Table 2. The first cost analysis of MSU was from trial-based research in Germany, which showed a favorable benefit-cost ratio between 2.16–6.85 at an operating distance between 26.73 and 40.32 miles, depending on the staffing (27). Another estimation was from Phantom-S study in Berlin (28), Germany, a 21-month multicenter RCT involving 28 hospitals, which found that the annual net cost of MSU deployment ($947,767) was balanced by an expected health benefit of 29.7 quality-adjusted life years (QALYs) per year. This calculation produced the incremental cost-effectiveness ratio of $31,911/QALY (Based on 2014, using cpi to remove inflationary effects). Kim et al. (29) conducted a model-based economic evaluation of MSU, obtaining data from MSU missions in Melbourne, Australia. This calculation concluded that deployment of MSUs avoided 27.94 DALYs and cost an additional $38,731 per DALY avoided. The recent economic evaluation is based on data obtained from the US Bureau of Labor and Statistics and peer-reviewed published literature, focusing on analyzing the incremental costs of MSU during the transport of patients. The model output was that use of MSUs cost an additional $70,613 per year, which would avoid 76 secondary transfers and 76 ED encounters (30).

## Discussion

The study assesses various outcomes of MSU in patients with acute stroke, explicitly focusing on the time metrics adverse events, long-term functional outcome, and cost-effectiveness. Twelve clinical studies reporting the previous MSU clinical trials and four economic research presenting related costs and benefits between 2012 and 2021 were included. Although pooled data from clinical studies demonstrated the significant time-saving effect of MSU, considerable heterogeneity exists between studies. It was probably a result of the different ranges of MSU deployment among studies [e.g., Walter et al. (21) reported the distance from a base station to the scene as 30 km as compared to approximately 10 km in Ebinger et al. (9) and 6.6 km in Helwig et al. (19)]. Furthermore, that stroke-related or neurological events and in-hospital mortality were not significantly improved is consistent with the previous meta-analysis (11). Next, the long-term outcome of mRs at 90 days from onset favored the MSU group, too. To consider the settings of RCTs the baseline mRs are similar between groups. In this way, the effect of MSU on reducing disability could be a reasonable extrapolation. Given that Prehospital time-saving may improve long-term outcomes, a couple of longitudinal researches are being prepared (31, 32). It should be informative to discuss the distance between potential stroke patients and the stroke unit. There are regional differences between countries, with stroke units sometimes very far away. MSU may be more effective regarding time to treatment and mortality in provincial cities and rural areas than in metropolises (33). However, the locations were primarily in urban instead of rural areas, except BEST MSU trial (22), thus raising many concerns about different outcomes for MSU in rural settings.

Four economic studies were conducted in three different countries. Despite inherent differences in health care systems, the MSU is a more expensive intervention than the EMS for acute stroke management in all selected countries. Therefore, the fundamental question is whether or not the MSU offers enough benefit to offset the additional costs incurred within the treatment. The present study's data show that despite the higher costs of the MSU, it creates upper QALYs and DALYs for patients compared with EMS. In the studies that present QALYs and DALYs indicators, MSU has generated higher values, inferring that the MSU program increased life expectancy, quality of life, and mRS in the long-term.

### Study Limitations

Our study has several limitations. First, data acquisition may vary by caregiver, so we cannot exclude information bias. Second, except for Grotta et al., studies did not adequately document the time to alarm and arrival at the scene regarding stroke so we may have overestimated the time saved for patients treated in the MSU. Third, most studies reported clinical outcomes for a maximum of 90 days from onset, which lacks long-term data to warrant the conclusion. Furthermore, the benefits that MSU provides to patients are obvious, but it is unclear what the national health service is willing to pay. In the absence of clear indicators of value, it is questionable to interpret conclusions about cost-effectiveness from the perspective of medical decision-making.

## Conclusion

In conclusion, a comprehensive analysis of current research on MSU indicates that this acute stroke management strategy is a better choice in various economic and social settings in terms of time metrics, safety, long-term medical benefits, and cost-effectiveness.

## Data Availability Statement

The original contributions presented in the study are included in the article/supplementary material, further inquiries can be directed to the corresponding author/s.

## Author Contributions

JC: methodology, formal analysis, and writing—original draft. XL and GZ: methodology and writing—review and editing. RH: writing—review and editing. YC: conceptualization, resources, and writing—review and editing. All authors contributed to the article and approved the submitted version.

## Conflict of Interest

The authors declare that the research was conducted in the absence of any commercial or financial relationships that could be construed as a potential conflict of interest.

## Publisher's Note

All claims expressed in this article are solely those of the authors and do not necessarily represent those of their affiliated organizations, or those of the publisher, the editors and the reviewers. Any product that may be evaluated in this article, or claim that may be made by its manufacturer, is not guaranteed or endorsed by the publisher.
